# Quantitative assessment of breast mammographic density with a new objective method

**Published:** 2011-08-25

**Authors:** G Iatrakis, S Zervoudis, E Sparaggis, P Peitsidis, P Economidis, P Malakassis, I Navrozoglou

**Affiliations:** * Technological Educational Institution of Athens, AthensGreece; ** Lito Hospital, Breast Department (Dept), AthensGreece; *** University of Ioannina, Gynecology Dept, Breast Unit, Ioannina Greece

**Keywords:** mammographic density, digital mammography, breast density assessment

## Abstract

Women with increased mammographic density (MD) have an increased risk of developing breast cancer. The purpose of our study is to evaluate an experimental method to quantify MD using a program (compatible with Windows XP, Vista and 7) which measures black areas as 0, white areas as 100 and grey scale areas with intermediate values between 0 and 100, depending on the “density” of the area. Digital screening mammograms were directly estimated with this method. Initial idea and steps of the program were based on a Mac utility used by our research team.

## Introduction

Digital mammography has been shown to have at least equivalent diagnostic accuracy to screen–film mammography and it offers some potential advantages over conventional technology [1] as magnification, subtraction of parasite signals, contrast and brightness changing, reproductivity and storage. In addition, digital mammography can be used for subjective measurement of breast density, which is a risk factor for future breast cancer. Studies reported in the literature indicate that the increase in the breast density is one of the strongest indicators of developing breast cancer [2, 3, 4]. Actually, women with high breast density are at higher risk of breast cancer and have larger screen–detected and interval cancers in mammographic screening programmes [5]. 

A set of fibroglandular density descriptors may be used within the text of a mammogram report, roughly corresponding to mammographic percent density (MPD) as: almost entirely fat, scattered fibroglandular densities, heterogeneously dense and extremely dense, corresponding to ≤25%, 26–50%, 51–75% and ≥76% MPD respectively [6]. Women with mammographic percent density (MPD) >50% have an approximately three–fold increased risk of developing breast cancer [7]. Similarly, the incidence of breast cancer among women with almost entirely dense breasts is three to sixfold greater than that of women with almost entirely fatty breasts, a relative risk approaching that conferred by a diagnosis of atypical ductal hyperplasia [8, 9, 10]. 

However, although breast density is a strong risk factor for breast cancer, no standard assessment method of MPD exists and some investigators presented their own method of density estimation [2, 11, 12].

The purpose of this work is to evaluate an experimental method to quantify MPD using a program (compatible with Windows XP, Vista and 7) specifically designed to calculate degrees of gray in mammographies

## Material and Methods

We realized a prospective study about a method of calculation of breast mammography density. Our main purpose was to obtain an objective value of mammographic density for digital mammography and to ‘avoid’ the subjectivity of estimation. 

**Figure 1:**
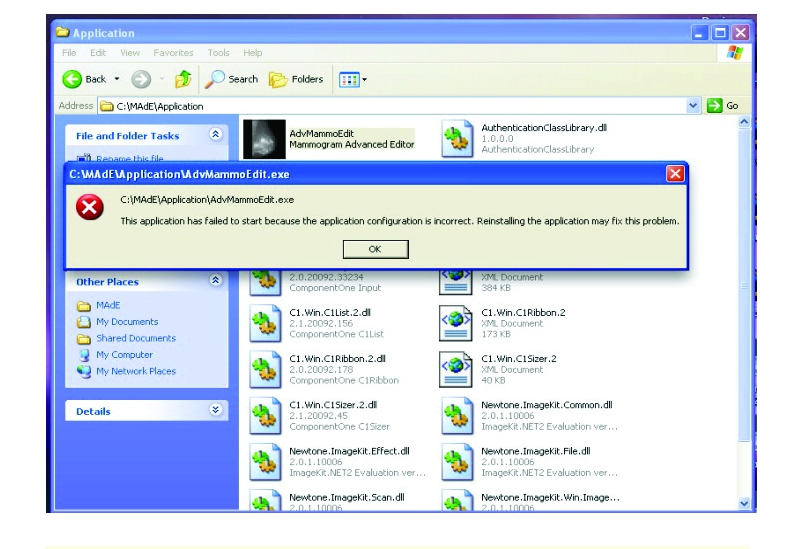
Initial incompatibility of the program with Windows XP

A special software (compatible with Windows XP, Vista and 7), which can quantify MD, was used the estimate breast density in digital mammographies. Initial version of the program was not compatible with Windows XP (Figure 1), a problem solved in more recent versions. 

The program ‘ignores’ absolutely black areas. In absolutely white areas, program estimation corresponds to one hundred. In grey scale areas the estimated values are between 0 and 100, depending on the ‘density’ of the area. 

**Figure 2:**
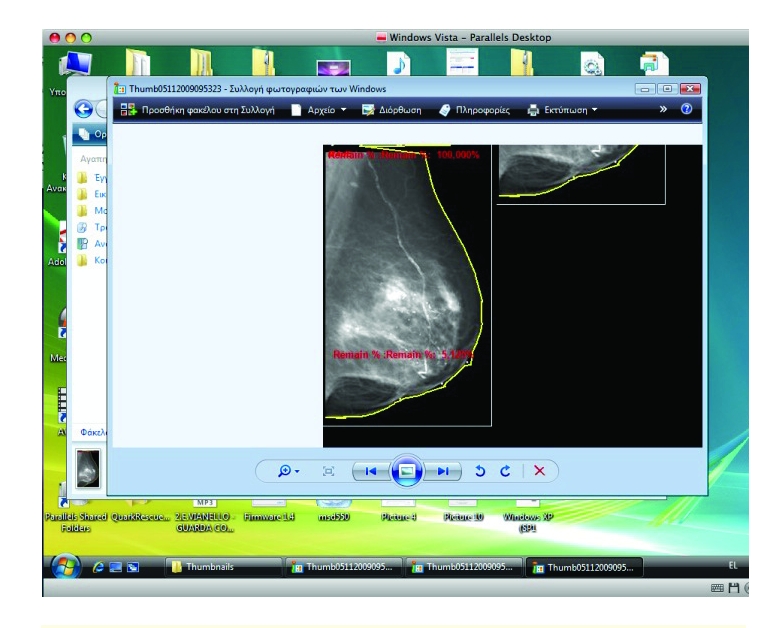
‘Surrounding’ the breast on the mammogram and measuring breast density in this particular area (compatibility with Windows Vista and Mac [Parallel]).

**Figure 3:**
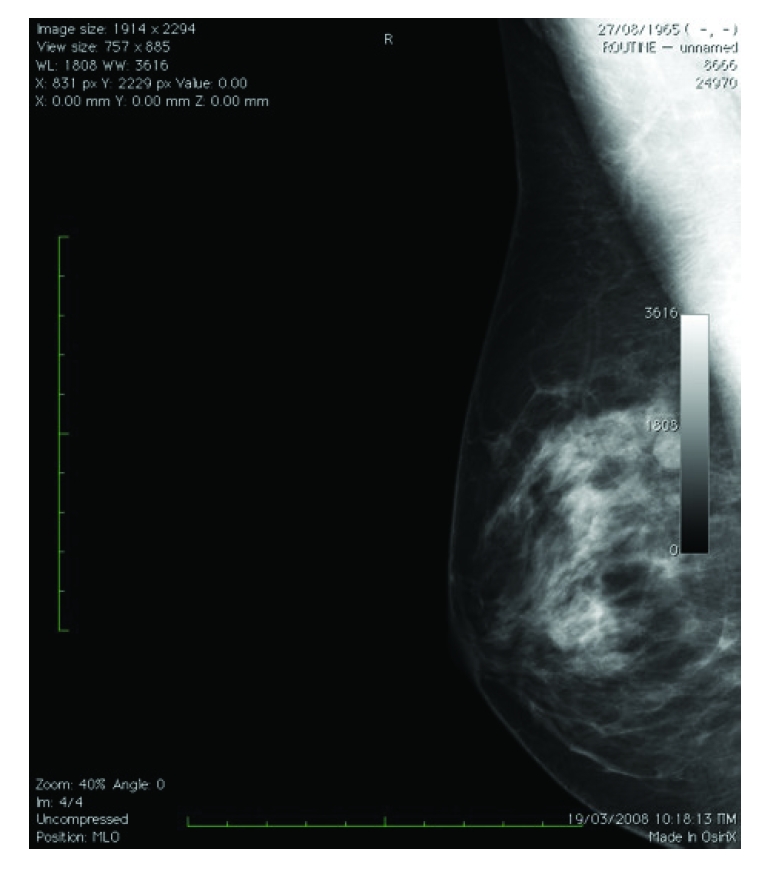
For reliable estimation of breast density only mammograms with good contrast must be used

**Figure 4:**
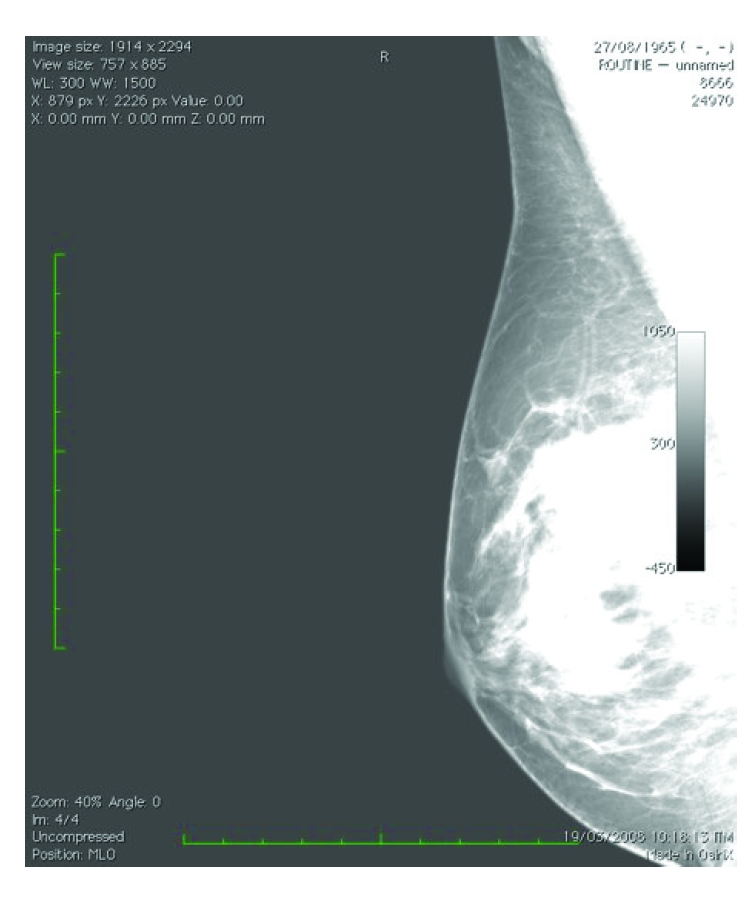
Poοr quality of the mammogram can result in overestimation of breast density

Moreover, the program has the capacity to ‘surround’ the breast on the mammogram and actually to measure breast density in this particular area (Figure 2). Initial idea, modifications and improvements of the program were suggested by the first author to the programmer. Further improvements related to microcalcifications estimation and ‘specialization’ of the program to recognize digital or digitized mammograms only were suggested by the second and first author respectively. For reliable measures, only mammograms with good ‘contrast’ can be used (Figure 3). Poοr quality of the mammogram can result in overestimation (Figure 4) or subestimation of breast density. 

Digital screening mammograms from 39 patients were directly estimated with the program and compared with clinical impression, blindly estimated by the authors. 

## Results

Density values in mediolateraloblique mammograms were increased in comparison with craniocaudal projections, due to the major pectoralis muscle (MPM). Therefore, to avoid the ‘whiteness’ of MPM, a special ‘intrument’ of the software was used which removes the MPM, which is replaced by an absolutely black area (ΑΒΑ). Taking into account that the program ignores ABA, this area is not calculated anymore in the final estimation of breast density. Similarly, the black background of mammograms (circumferentially of the breast) did not lower the real density value in mammograms, because as already mentioned, ABΑ is not calculated in the final estimation. In cases of ‘grey’ background, mammograms' contrast changed until to achieve an ABΑ background. Hence, only mammograms with correct contrast included in the final results. This method assured the correct density comparison among mammograms because the same scale of contrast was used to all mammograms and the breast itself had the correct mammographic ‘density’. Equally, with this method, no further calculations were necessary to compare breast densities among mammograms or the use of special tables, and the ‘automated’ percentages corresponded to the real mammographic density of a particular breast. 

In relation to clinical impression, we found a significant correlation between ACR quartiles, clinically estimated, with this grey scale percentage method. However, a significant percentage of clinical estimations (18%) could be classified as ‘not accurate’ with a discrepancy more than 20% of the ‘exact’ percentage of breast density. In particular, 7 mammogram densities were over– or subestimated (> 20 to 27% or > –20 to –25%). 

## Discussion

Mammographic screening usually involves the performance of the mediolateraloblique and craniocaudal projections. In a previous publication of our research team, due to the square shape of the aperture area examined with a Mac utility, MPM was not possible to be avoided without lose a part of breast tissue in mediolateraloblique projections. On the contrary, with this specially constructed program, the MPM was removed in all mediolateraloblique mammograms and this area was not calculated in the final estimation of breast density.

Subjective fibroglandular density description could be a useful tool of breast cancer risk estimation in a particular woman. However, some degree of hesitation could arise when such a description belongs to MPD with increased risk of breast cancer. On the contrary, our method has ‘descriptive accuracy’. The method could be proposed as an objective tool of breast density measurement, a comparative tool between mammograms of the same woman in different periods with different stimulating factors of mammary proliferation and as comparative tool among mammograms of different women, subgrouping them in age groups, treatment groups etc. Similarly, previous studies, using a variety of methods, quantified objectively the MPD, correlate it with breast cancer risk and made digitized assessments of mammographic breast density in patients receiving hormonal regimens [13, 14, 15]. However, some of them is more difficult to understand and/or more time cossuming. 

Taking into account that breast density is actively related to breast cancer risk, methods of breast densitometry must be accurate, reliable, easy to learn, easy to perform, widely available, quick, cheap and repeatable. 

We believe that this method consists an essential improvement of our previous one regarding to accuracy and estimation of breast density in the exact ‘shape’ of a particular breast. 
